# Quantum Vibrational Spectroscopy of Explicitly Solvated Thymidine in Semiclassical Approximation

**DOI:** 10.1021/acs.jpclett.1c04087

**Published:** 2022-02-03

**Authors:** Fabio Gabas, Riccardo Conte, Michele Ceotto

**Affiliations:** Dipartimento di Chimica, Università degli Studi di Milano, via Golgi 19, 20133 Milano, Italy

## Abstract

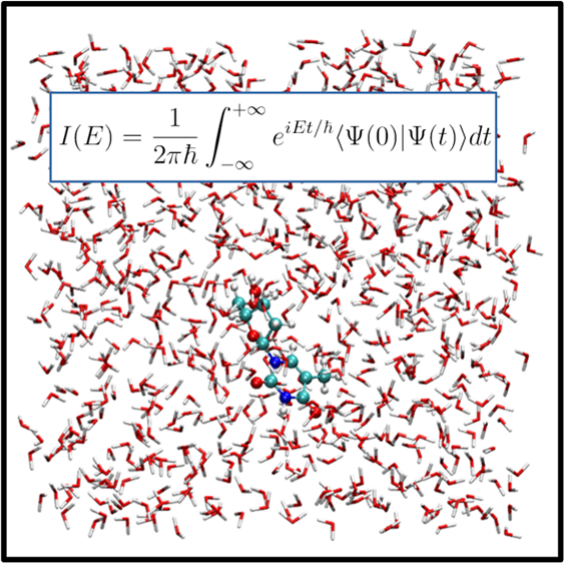

In this paper, we demonstrate the possibility to perform spectroscopy simulations of solvated biological species taking into consideration quantum effects and explicit solvation. We achieve this goal by interfacing our recently developed divide-and-conquer approach for semiclassical initial value representation molecular dynamics with the polarizable AMOEBABIO18 force field. The method is applied to the study of solvation of the thymidine nucleoside in two different polar solvents, water and *N*,*N*-dimethylformamide. Such systems are made of up to 2476 atoms. Experimental evidence concerning the different behavior of thymidine in the two solvents is well reproduced by our study, even though quantitative estimates are hampered by the limited accuracy of the classical force field employed. Overall, this study shows that semiclassically approximate quantum dynamical studies of explicitly solvated biological systems are both computationally affordable and insightful.

In contemporary science, there is great effort to address and fully understand the molecular properties of constituent fragments of DNA and RNA. The importance of such building blocks of life lies mainly in the fact that even a minor modification in their molecular structure can largely affect their biological functionality.^1^ Therefore, nucleobases and nucleosides, with the latter differing from the former owing to the presence of an additional five-membered furanose ring, have been largely studied both experimentally and theoretically. Specifically, the possibility to simulate such systems in their natural environment, i.e., water, is crucial to understand their behavior in vivo.

It is well-known that water solvation has a great influence in the majority of biological processes ranging from cellular function to biomolecular interactions, from biopolymer stability to solvation of simple solutes.^2−5^ In such systems, water is not a simple passive medium but has a leading role.^6^ When polar solutes are involved, for example, solvation takes place engaging the dipole moment of water that reorients itself in response to the solute charge distribution. Remarkably, it has been estimated that electronic solvation could account for up to half of the overall solvation free energy.^6^ Even in the presence of hydrophobic solutes, water behavior is active and plays an important role, for instance, in the hydrophobic effect. Several works, based on different experimental techniques such as nuclear magnetic resonance (NMR), high-performance liquid chromatography (HPLC), and neutron scattering, show that, in the presence of an apolar solute, water rearranges its hydrogen bond network creating a cavity.^7−10^ Interactions between the solute and the solvent, for nonpolar solvation, derive mainly from weak dispersion forces originating from the fluctuation of induced dipoles within solvent and solute molecules rather than from the electrostatics of charge distributions as in the polar solutes case.^11−15^ Furthermore, water can also interact with solutes in a site-specific and “nonbulk” manner. Nucleic acids offer a good example of how water can interact in a sequence-specific manner, such as the zigzag spine of hydration in the minor groove of B-DNA.^3,16−20^

For all these reasons, when these kinds of systems have to be computationally simulated, an explicit treatment of the solvent should be in principle preferred over an implicit one. However, simulating biological molecules using explicit water molecules requires high computational costs and resources. These requirements entirely exclude the possibility of using accurate ab initio molecular dynamics methods (AIMD), in which the potential energy is evaluated adopting an ab initio quantum method, such as MP2, Coupled Cluster, or the family of DFT functionals. Therefore, the most common type of potential employed in explicit solvation is obtained from classical molecular mechanics (MM) carried out by using popular and well-tested force fields, such as AMBER,^21,22^ CHARMM,^23,24^ or AMOEBABIO18.^25,26^ This approach permits even dynamical studies of large dimensional systems, because the quantum electronic structure is not calculated and polarization effects are quite approximated. To recover a more accurate chemical description, the hybrid quantum mechanical/molecular mechanics method (QM/MM) is a feasible option, often used to treat the electronic problem in case of chemical reactions or enzymatic processes in condensed phase.^27−30^ In such an approach, the total system is partitioned into subsystems treated with different theories according to the level of detail needed. QM/MM represents a good compromise between feasibility and chemical accuracy, but it presents some difficulties such as domain partitioning and dynamical continuity between the partitions.^27^

In this work, we employ the affordable molecular mechanics description of the potential, through the polarizable AMOEBABIO18 force field, with the aim to investigate the vibrational power spectrum of thymidine in explicit solvents. Specifically, the general importance of thymidine lies in the fact that some of its analogues were intensively employed as anti-HIV drugs. In the literature, several studies involving experimental work about thymidine in water and the theoretical analysis of thymidine interacting with one or more water molecules are found.^31−35^ To recover a high level of chemical description in detecting the vibrational spectrum, we use the divide-and-conquer semiclassical initial value representation (DC SCIVR) method, which relies on classical trajectories but is able to provide quantum effects by means of the semiclassical formalism.^36−38^ Indeed, one of the strengths of semiclassical dynamics (and the DC-SCIVR approach) lies in its capability to include all kinds of quantum effects like anharmonic overtones and combination bands, in addition to the anharmonic contributions of the potential energy surface.^39−48^ By choosing this method and by accounting for the water solvent explicitly, we aim at reproducing solvation in a remarkably accurate and complete way.

In this context, after investigating the spectroscopy of nucleobases,^49^ we recently presented a study that involves four nucleosides.^50^ In that paper we showed and discussed their vibrational power spectra in gas phase, obtained by means of the semiclassical DC-SCIVR method performed with AMOEBABIO18 calculations. In that analysis, we compared the AMOEBABIO18 performance against ab initio DFT calculations in reproducing experimental vibrational frequencies by means of DC SCIVR. This allowed us to point out the reasonable quality of the predictions obtained with that force field with respect to the perhaps more popular AMBER competitor. Furthermore, we presented the power spectrum of thymidine in the range of 1500–1800 wavenumbers, where some important nucleobase spectral signatures are located. In particular, we could analyze the C4O and C5C6 stretchings (see Figure 1 for the atom numbering employed) that are also detected by the gas phase experiment.^51^

**Figure 1:**
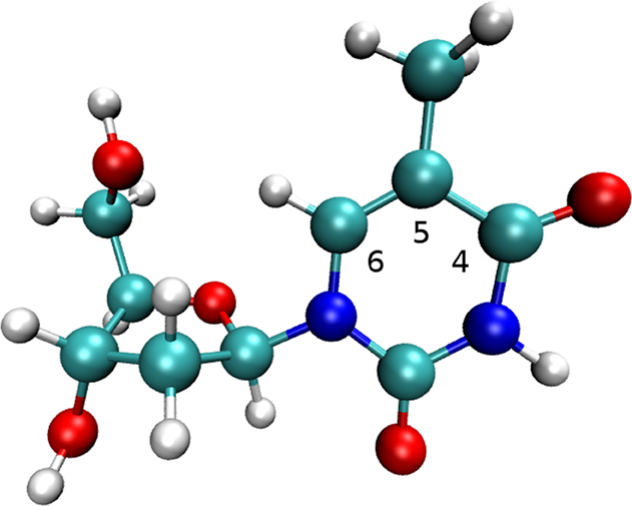
Relevant atomic numbering of isolated thymidine.

In the present work, the experimental frequencies we take as a reference for thymidine in condensed phase are instead offered by the research of Loppnow and co-workers.^33^ In their paper they showed a comparison between Raman spectra of thymine and thymidine recorded in the 750–1850 cm^–1^ frequency range employing different solvents, among which are water and *N*,*N*-dimethylformamide (*N*,*N*-DMF). They noticed that stretching signals associated with thymidine C4O and C5C6 bonds were basically degenerate at around 1710 cm^–1^ in the case of water and almost all other solvents, with the exception of *N*,*N*-DMF, in which case a double peak in the same frequency region was detected. This scenario offers us the possibility to demonstrate that the semiclassical DC-SCIVR method, together with AMOEBA force fields, can be efficiently employed for simulation of biological systems in condensed phase, reproducing at least qualitatively the correct effect of different solvents on the vibrational features of thymidine.

All the simulations have been performed using the AMOEBA force fields, as implemented in the Tinker 8.6.1 software. In particular, we present our studies of thymidine in water, as parametrized in the AMOEBABIO18 force field, while we used AMOEBA09 parameters to model the *N*,*N*-DMF solvent, since it is not present in AMOEBABIO18.^35,52^ For both solvents, we inserted the thymidine molecules in a cubic box whose sides measured 30 Å, as represented in Figure 2. In all calculations, the periodic boundary conditions are fully considered, using the particle mesh Ewald (PME) scheme for the long-range interactions.^53,54^

**Figure 2:**
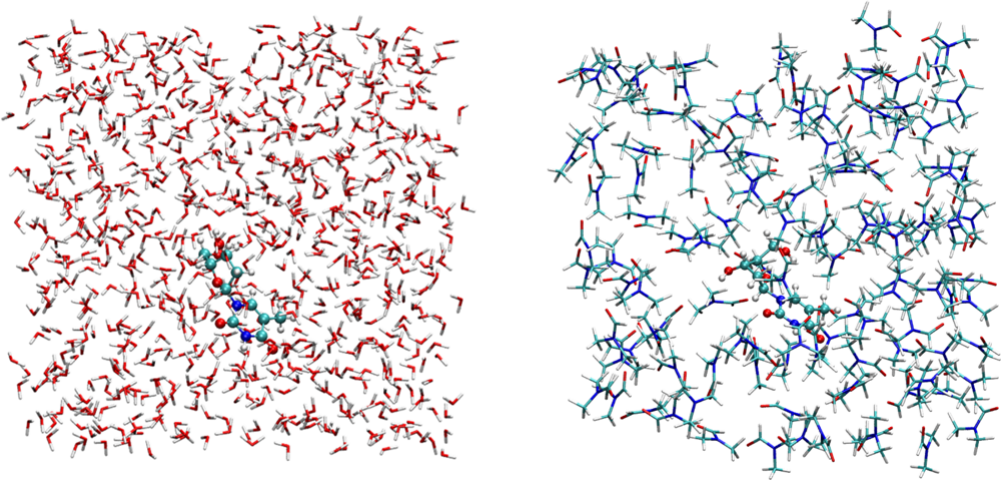
Thymidine in a box of water (left) and *N*,*N*-DMF (right) molecules.

The minimization and the subsequent Hessian matrix calculation led us to estimate the harmonic vibrational frequencies. After the optimization phase, NVE trajectories have been propagated using periodic boundary conditions and Beeman’s integration algorithm for a total time of 0.6 ps, employing a time step equal to 0.2 fs. To produce the spectra, we adopted the DC-SCIVR theoretical method. Such a methodology has its foundation in the earlier time averaged (TA-SCIVR) and multiple coherent (MC-SCIVR) semiclassical initial value representation approaches, and it can be fruitfully applied to large dimensional systems thanks to the projection of all quantities involved onto subsystems of reduced dimensionality.^36,55−63^ The DC-SCIVR working formula we employed is
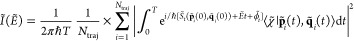
1where  are the projected configurations and momenta of the system degrees of freedom at the beginning of the trajectory, *T* is the total simulation time,  is the instantaneously projected classical action at time *t*,  is the Fourier-transform projected energy, and  is the phase of the projected prefactor, whose definition is
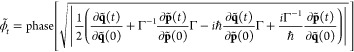
2Such an approach requires evaluation of the Hessian matrix step by step along the trajectory and the potential projected in the subspace, which contributes to the evaluation of . Following a well-consolidated protocol of semiclassical spectroscopy,^37,59^ we employed a single trajectory characterized by a harmonic estimate of the NVE trajectory energy. Among all the existing procedures to partition the total degrees of freedom in subspaces, we chose the average Hessian matrix criterion,^61^ which led to monodimensional subspaces for the C5C6 and C4O stretchings under examination for both solvents. We adopted the Hessian update approximation by evaluating only one Hessian matrix every 100 dynamics steps and using the gradient to estimate it for the other steps.^64,65^ The rationale behind this choice is that the approximation has been demonstrated in several applications to be accurate enough^65^ and the amount of data that 3000 Hessian matrix calculations produce would be too high to be stored, in spite of the limited computational cost required for Hessian matrix calculations with AMOEBABIO18. For the simulation using water as the solvent, we chose the zero point energy (ZPE) trajectory. This is a trajectory in which every normal mode has a starting velocity derived from the estimated harmonic ZPE energy. For the simulation of thymidine in *N*,*N*-DMF solvent, one trajectory was run for each mode under investigation and, to better account for the coupling between modes, the trajectory was prepared in a different way as successfully tested in previous applications of semiclassical dynamics.^66−69^ Specifically, atomic velocities were determined such that the total energy included a quantum of excitation in the vibrational mode of thymidine under investigation in addition to the ZPE energy, while the starting geometry was slightly displaced from the equilibrium one. Details can be found in the Supporting Information.

All the calculated frequencies are reported in Table 1, while Figure 3 shows the comparison between the experimental findings and the AMOEBABIO18 DC-SCIVR spectra. The harmonic frequencies are reported in the figure with vertical sticks positioned above the semiclassical peaks. As already discussed in our previous work,^50^ AMOEBABIO18 can be successfully employed to obtain qualitative results. For this reason, we started looking at gaps between spectral signals rather than single frequency values. In fact, the isolated thymidine C5C6 and C4O stretching frequencies reported in our previous paper highlight the correct gap between these stretchings, equal to 70 cm^–1^, to be compared with the gas-phase experiment which reports a gap equal to 52 cm^–1^. We obtained similarly reliable results upon moving to solvated systems. Figure 3 shows that when thymidine is solvated by water, the two semiclassical fundamental peaks for C5C6 and C4O are very close to each other, being only 30 cm^–1^ apart. When *N*,N*-*DMF is employed as a solvent, instead, we expect a different picture because the experiment displays a clear double peak. This feature is well reproduced in our DC-SCIVR calculations, which indicate a 70 cm^–1^ gap in this case. It is important to note that the simple harmonic calculation is not able to regain the same difference, giving for both solvents a gap approximately equal to 50 cm^–1^.^33^ Furthermore, in the case of *N*,*N*-DMF, the investigated thymidine peaks appear much less split than in water, since fewer interactions influencing them are present. To further prove that, we notice that the semiclassical spectra of thymidine in water present more than one pronounced peak around the fundamental one, in particular for the C4O signal. They are present because of all the interactions that affect the C4O stretch, which come from the solvent but also from other thymidine modes of vibrations like the C5C6 stretch. We recall that we are calculating the power spectrum, i.e., the vibrational density of states, and there are several vibrational states coupled to the C4O stretch within the investigated energy range. In fact, the DC-SCIVR methodology is able to recover overtones and combination bands, which certainly contribute to enrich the vibrational spectrum.

**Table 1:** Experimental, Harmonic, and Semiclassical Vibrational Frequencies for All the Systems Investigated^a^

	expt	harm	DC-SCIVR
isolated thymidine^50^			
C4O	1714	1688	1670
C5C6	1662	1578	1600
Δ	52	110	70
thymidine in water			
C4O	∼1710	1657	1597
C5C6	∼1710	1611	1567
Δ	0	46	30
thymidine in *N*,*N*-DMF			
C4O	∼1700	1650	1640
C5C6	∼1620	1606	1570
Δ	80	44	70

a Δ is the difference between the C4O and C5C6 stretching frequencies.

**Figure 3:**
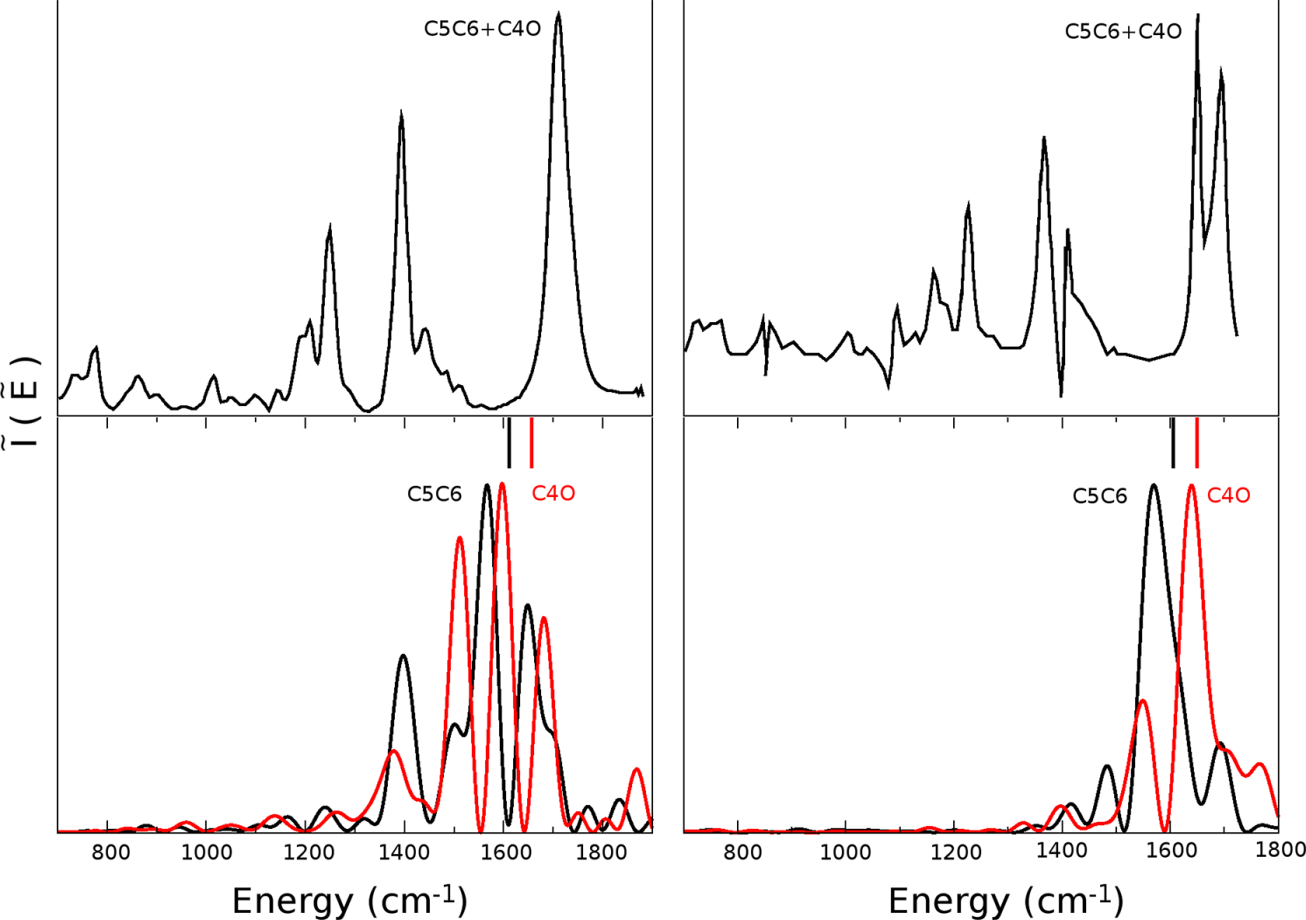
Comparison between semiclassical spectra of thymidine in a box of water (left) and *N*,*N*-DMF (right) and the results of Loppnow’s Raman experiment.

Moving to the analysis of the frequencies of vibration, we observe that the harmonic estimates obtained with AMOEBABIO18 are lower than the experimental frequencies for both simulated solvents, as it was in our previous work.^50^ Our anharmonic method returns even lower frequencies. Indeed, the DC-SCIVR prediction of the two frequencies in water is 1567 and 1597 cm^–1^ for the C5C6 and C4O normal modes, respectively, while the experiment highlights a single peak positioned at around 1710 cm^–1^. Due to the intrinsic accuracy of our method, this result for the closing gap is consistent with the experimental finding and much smaller than the gap for isolated thymidine (70 cm^–1^). Moving to the *N*,*N*-DMF solvent, we obtained 1570 and 1640 cm^–1^, once again for C5C6 and C4O, respectively. This should be compared to the experiment having the two signals roughly located at 1620 and 1700 cm^–1^ respectively. Therefore, the gap is correctly described, definitely larger than the one found in water, but the frequencies are somewhat shifted with respect to the experiment. However, we are not interested in reproducing exactly peak positions, a task which is not feasible due to the limited accuracy of the employed force fields and would require a higher level potential energy surface, but their behavior under different conditions. In particular, we want to demonstrate that our approach, combined with AMOEBA force fields, is able to reliably reproduce the effect that different solvents can have on a solvated molecule. Finally, there is one aspect in which our calculations somewhat differ from the experiment: while experimentally the closing gap in water is due to the blue shift of the C5C6 frequency, in our simulations it is the C4O frequency which strongly red shifts. The force field is overestimating the interaction between the C4O lone pair and water hydrogens generating the red shift. Indeed, this issue disappears in the simulation for *N*,*N*-DMF, which is not characterized by the same hydrogen bond network as water.

In this work, we applied our DC-SCIVR method for the first time to study a condensed phase system of biological interest. The limited computational cost required by AMOEBA force fields allowed us to study the thymidine nucleoside in two different solvents, water and *N*,*N*-DMF, in an explicit fashion, and even adopting periodic boundary conditions to mimic a complete molecular solvation. Our DC-SCIVR method, in conjunction with AMOEBABIO18, confirms its capability to reproduce qualitatively the experimental observations at the quantum mechanical level. Even if a quantitative prediction is not possible due to the limited accuracy of AMOEBABIO18, qualitative studies may open the route to vibrational studies of solvated biological molecules, bringing the community to a deeper understanding of structures and properties of biomolecules.

As a final remark, it should be noticed that in AMOEBABIO18 quantum effects are included in a meanfield way via the force field parametrization to directly reproduce a list of experimental condensed phase properties. Therefore, the force field is designed for classical simulations and performing quantum (semiclassical) calculations could lead to a “double counting” of quantum effects deteriorating the accuracy of the results. While this issue may affect the simulation of vibrational modes involving mainly hydrogen atoms (for instance, high frequency C–H, N–H, and O–H stretches) this is not the case for the simulations presented here.

## Supporting Information Available

Details about the setup of simulations and the force field parameters employed (PDF)
Transparent Peer Review report available (PDF)
