# Supplementary material for: Quantum Vibrational Spectroscopy of Explicitly Solvated Thymidine in Semiclassical Approximation
Source: J Phys Chem Lett. 2022 Feb 3;13(5):1350–5. doi: 10.1021/acs.jpclett.1c04087 (PMC8842300; doi:10.1021/acs.jpclett.1c04087)
Supplement: Supplementary file 1 — jz1c04087_si_001.pdf [file jz1c04087_si_001.pdf]

# Quantum Vibrational Spectroscopy of Explicitly Solvated Thymidine in Semiclassical Approximation

Fabio Gabas, Riccardo Conte, and Michele Ceotto*

Dipartimento di Chimica, Università degli Studi di Milano, via Golgi 19, 20133, Milano, Italy

E-mail: michele.ceotto@unimi.it

All the semiclassical spectra presented in the main article have been produced following the standard MC-DC-SCIVR procedure:

1. system preparation and setup;
2. optimization and harmonic frequencies calculation;
3. initial conditions preparation (i.e. starting velocities and positions in cartesian coordinates);
4. short-time dynamics trajectory run;
5. Hessian matrices along the trajectory calculation;
6. division of the degrees of freedom in subspaces of reduced dimensionality;
7. evaluation of potential energy along the trajectory, projected in the subspace of interest;
8. evaluation of the vibrational spectrum.

We employed the AMOEBABIO18 force field as implemented in the Tinker 8.6.0 suite. Such package handles every task through a different software but requiring always for a keyfile containing the list of all the parameters adopted. These are reported in Figure S1.

```
parameters amoebabio18.prm
digits 8
verbose
a-axis 30.0
bondtype morse
cutoff 9.0              # Nonbonded interactions direct cutoff
ewald                   # Switch on PME
ewald-cutoff 7.0        # PME real space cutoff (overrides the 9A above)
vdw-correction          # Switch on analytical long-range vdW correction
polar-eps 0.01          # Dipole convergence criterion in RMS Debye/atm
neighbor-list           # Use pairwise neighbor list for calculating nonbonded interactions (improves speed)
```

Figure S1: Base parameter set used for AMOEBABIO18 simulations with the Tinker package.

The first three rows provide the code with the force field parameter file, enable the use of 8 digits for the numeric precision and set a high grade of informations printing in the output file. 'a-axis 30.0' sets to 30 angstrom the value of the a-axis length for the periodic box. In absence of specific parameters for b and c axis, they are set at the same length of the a-axis. The Morse bondtype is chosen to properly model the bond terms of the force field energy expression. Then there is a list of parameters that defines how to manage the interaction calculations. We set to 9.0 Angstrom the cutoff for the non-bonded interactions and to 7.0 the cutoff for the real-space distance during Particle Mesh Ewald summation, enabled for the computation of partial charge, atomic multipole and polarization interactions in periodic systems.

The three last parameters help in improving the speed of the calculations. 'VdW correction' turns on the use of an isotropic long-range correction term to approximately account for van der Waals interactions beyond the cutoff distance, 'polar eps' set to 0.01 the dipole convergence criterion and 'neighbor-list' enable pairwise neighbor list for calculating nonbonded

interactions.

Additionally, we used specific parameters for each particular task of the semiclassical recipe. For the minimization step we add to the keyfile the following lines showed in Figure S2, while for the dynamics run we add the lines showed in Figure S3.

```
12)  maxiter 2000             # Maximum number of minimization steps
13)  steepest-descent         # Use the SD minimization algorithm
```

Figure S2: Other parameters used in addition to those listed in S1 for AMOEBABIO18 minimization with the Tinker package.

'Steepest-descent' is the minimization algorithm chosen to speed-up the calculation, with a maximum iteration number equal to 2000.

```
12)  save-velocity
13)  integrator Beeman
```

Figure S3: Other parameters used in addition to those listed in S1 for AMOEBABIO18 dynamics run with the Tinker package.

With the 'save-velocity' keyword it is possible to save in the output file the Cartesian velocities along the dynamics. It represents a mandatory requirement for the construction of the DC-SCIVR spectrum. 'Integrator Beeman' sets to Beeman the select method of dynamics trajectory propagation. The subspaces partition has been performed by using the Hessian averaged criterion, over the 30 Hessian matrices calculated along the trajectories. In all the cases this criterion led to monodimensional subspace.
