# Supplementary material for: Quantum Vibrational Spectroscopy of Explicitly Solvated Thymidine in Semiclassical Approximation
Source: J Phys Chem Lett. 2022 Feb 3;13(5):1350–5. doi: 10.1021/acs.jpclett.1c04087 (PMC8842300; doi:10.1021/acs.jpclett.1c04087)
Supplement: Supplementary file 2 — jz1c04087_si_002.pdf [file jz1c04087_si_002.pdf]

jz-2021-040875.R1

Name: Peer Review Information for "Quantum Vibrational Spectroscopy of Explicitly Solvated Thymidine in Semiclassical Approximation"

First Round of Reviewer Comments

Reviewer: 1

Comments to the Author

The authors give a very good discussion and summary of the complex dynamical effects a solvent can have on a solute. They also discuss the ability of various versions of the semiclassical initial value representation (SCIVR) to provide a good description of quantum dynamics, by the calculation of various time correlation functions (dipole-dipole for spectra, flux-flux for reaction rates, etc.); this has been verified earlier by a number of applications to relatively simple molecular systems.

The challenge has been to develop methods for the practical implementation of SCIVR approaches to very complex molecular systems, such as those treated in this paper. In this regard the Milan group has made very impressive progress. At this point the weakest link in the ability of theory to provide quantitative results seems to be inaccuracy of the empirical potential energy surfaces. Being able to implement the SCIVR approaches with ab initio potential surfaces is obviously the next hurdle to overcome.

The most encouraging aspect of the. present calculations (in Table 1) is that the SCIVR results for the splitting of stretching frequencies (the Delta result) is noticeably better than the harmonic results (though the absolute frequencies are not particularly so).

Finally, one wonders if the results would be better if the calculation was for the relevant time-correlation function (e.g., dipole-dipole) were calculated rather than the 'power spectrum' as done here. This would of course require knowing the dipole moment of the system as a function of molecular coordinates.

In summary, I can recommend the paper for publication, even though there are still areas in the methodology that can be improved.

Reviewer: 2

Comments to the Author

In this paper Gabas et al. interfaced their recently developed divide-and-conquer approach for semiclassical initial value representation molecular dynamics with the polarizable

AMOEBABIO18 force field in order to perform spectroscopy simulations of solvated biological species taking into consideration quantum effects and explicit solvation. The chosen test system is the solvated thymidine nucleoside in two different polar solvents: water and N,Ndimethylformamide. Experimental data appear to be qualitatively well reproduced.

The paper is well written and informative but needs to adress a major point that is currently missing.

Indeed, the authors conclude that "quantitative estimates are hampered by the limited accuracy of the classical force field employed" however, such force field is designed to be use in a classical molecular dynamics simulations. Indeed, in AMOEBA, no explicite quantum nuclear effects are present as they are simply included in a meanfield way via the force field parametrization to directly reproduce a list of experimental condensed phase properties (therefore including all types of effects). Here, the force field is NOT used in its normal context since this study employs semiclassically approximate quantum dynamics and not classical dynamics. What about double countings of quantum nuclear effects? Nothing is said about them: they could be very large source of errors. The authors should dig up in that direction and study the potential impact of combining a force field designed for condensed phase and their semi-classical approach.

Author's Response to Peer Review Comments:

Reviewer: 1

Comments:
The authors give a very good discussion and summary of the complex dynamical effects a solvent can have on a solute. They also discuss the ability of various versions of the semiclassical initial value representation (SCIVR) to provide a good description of quantum dynamics, by the calculation of various time correlation functions (dipole-dipole for spectra, flux-flux for reaction rates, etc.); this has been verified earlier by a number of applications to relatively simple molecular systems.

Our Reply:
We thank Reviewer 1 for her/his very positive assessment of our work. We generally agree with the comments, but we would like to clarify a couple of aspects.

The challenge has been to develop methods for the practical implementation of SCIVR approaches to very complex molecular systems, such as those treated in this paper. In this regard the Milan group has made very impressive progress. At this point the weakest link in the ability of theory to provide quantitative results seems to be inaccuracy of the empirical potential energy surfaces. Being able to implement the SCIVR approaches with ab initio potential surfaces is obviously the next hurdle to overcome.

Our Reply:
We have already demonstrated to be able to employ our semiclassical techniques with ab initio potential energy surfaces. Clearly the issue in the present case is that an ab initio potential energy surface for a complex system such as solvated thymidine is not available and it would be very complicated to build one. This is the reason why we had to rely on the AMOEBA force field.

The most encouraging aspect of the present calculations (in Table 1) is that the SCIVR results for the splitting of stretching frequencies (the Delta result) is noticeably better than the harmonic results (though the absolute frequencies are not particularly so).
Finally, one wonders if the results would be better if the calculation was for the relevant time-correlation function (e.g., dipole-dipole) were calculated rather than the 'power spectrum' as done here. This would of course require knowing the dipole moment of the system as a function of molecular coordinates.

Our Reply:
The suggestion to perform a dipole-dipole autocorrelation function is potentially a good one and would add information on the amplitudes of our spectroscopic signals. Actually our current development efforts are going in the direction suggested by the Reviewer. However, our calculations aimed at a comparison between calculated and experimental frequency gaps. For such an investigation the information contained in a power spectum is sufficient and a dipole-dipole based calculation is not expected to improve results significantly. Furthermore, a dipole estimate obtained from a force field would probably be not accurate enough, not allowing us to move from qualitative to quantitative estimates of the frequencies of vibration.

In summary, I can recommend the paper for publication, even though there are still areas in the methodology that can be improved.

Our Reply:
We thank the Reviewer again. We have not made any changes to our manuscript following her/his comments.

Reviewer: 2

Comments:
In this paper Gabas et al. interfaced their recently developed divide-and-conquer approach for semiclassical initial value representation molecular dynamics with the polarizable AMOEBABIO18 force field in order to perform spectroscopy simulations of solvated biological species taking into consideration quantum effects and explicit solvation. The chosen test system is the solvated thymidine nucleoside in two different polar solvents: water and N,Ndimethylformamide. Experimental data appear to be qualitatively well reproduced. The paper is well written and informative but needs to adress a major point that is currently missing.

Our Reply:
We thank the Reviewer for her/his positive assessment of our paper.

Indeed, the authors conclude that "quantitative estimates are hampered by the limited accuracy of the classical force field employed" however, such force field is designed to be use in a classical molecular dynamics simulations. Indeed, in AMOEBA, no explicite quantum nuclear effects are present as they are simply included in a meanfield way via the force field parametrization to directly reproduce a list of experimental condensed phase properties (therefore including all types of effects). Here, the force field is NOT used in its normal context since this study employs semiclassically approximate quantum dynamics and not classical dynamics. What about double countings of quantum nuclear effects? Nothing is said about them: they could be very large source of errors. The authors should dig up in that direction and study the potential impact of combining a force field designed for condensed phase and their semi-classical approach.

Our Reply:
The point raised by the Reviewer is a very important one. In the Figures below we show a comparison between semiclassical and quasi-classical trajectory (QCT) simulations based on the same trajectories. QCT is a classical molecular dynamics technique characterized by NVE trajectories at zero point energy shell. In this way, initial conditions are quantized. We notice that the frequency estimates (x-axis in cm^{-1}) provided by the two methods for the target modes are basically equivalent.

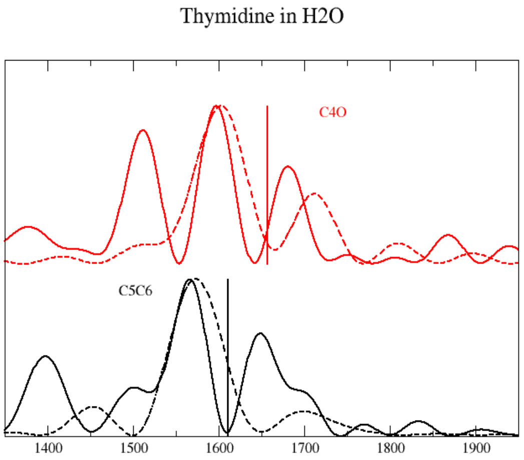

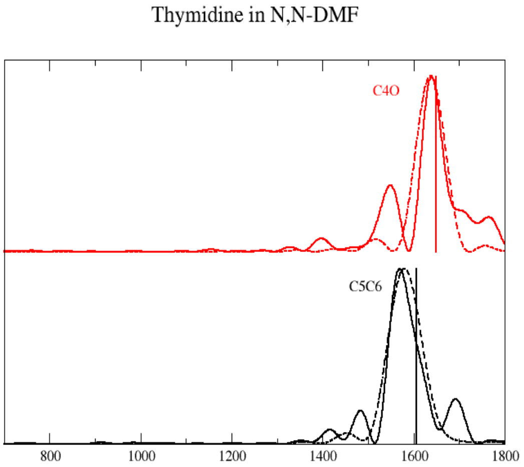

In the Figures above the QCT simulations are reported with dashed lines and the semiclassical ones with continuous lines. The vertical stikcks represent the harmonic estimates. The semiclassical spectra contain more spectral features because they are able to detect all quantum combination bands which are not present in the QCT spectra.

This finding corroborates our conclusion that, at least in the present case, it is the limited accuracy of the classical force field employed, not the semiclassical approach, which hampers the quantitative accuracy of our calculated frequencies with respect to the experiment. However, we stress that we found AMOEBABIO18 to be the most suitable force field for our purposes due to its description of polarizability (see Gabas et al, "Semiclassical vibrational spectroscopy of biological molecules using force fields", *JCTC* **16**, 3476 (2020)), and we have recognized this already in the original version of our manuscript.

The comment of the Reviewer could still be generally appropriate for vibrational modes (for instance high frequency stretches involving hydrogen atoms) in which quantum effects may be dominant (see for example Gabas et al. "Protonated glycine supramolecular systems: the need for quantum dynamics", *Chem. Sci.*, **,** 7894 (2018)). However, we point out that in this study we are interested in frequency differences (between two vibrational modes) which, being a relative value, are less sensitive to the issue pointed out by the Reviewer than absolute frequency values.

In conclusion, we agree with the Reviewer that the issue of double counting quantum effects may arise when using our semiclassical techniques with force fields, but it does not affect the conclusions of the current paper.

We have inserted the following short paragraph on the issue at the end of the revised version of the manuscript:

"*As a final remark, it should be noticed that in AMOEBABIO18 quantum effects are included in a meanfield way via the force field parametrization to directly reproduce a list of experimental condensed phase properties. Therefore, the force field is designed for classical simulations and performing quantum (semiclassical) calculations could lead to a "double counting" of quantum effects deteriorating the accuracy of results. While this issue may affect the simulation of vibrational modes involving mainly hydrogen atoms (for instance high frequency C-H, N-H, and O-H stretches) this is not the case for the simulations presented here.*"
